# Uncoupling of Ca^2+^ sparks from BK channels in cerebral arteries underlies hypoperfusion in hypertension-induced vascular dementia

**DOI:** 10.1073/pnas.2307513120

**Published:** 2023-08-07

**Authors:** Jade L. Taylor, Katy R. Walsh, Ioana-Emilia Mosneag, Thea G. E. Danby, Nadim Luka, Bishal Chanda, Ingo Schiessl, Ross A. Dunne, David Hill-Eubanks, Grant W. Hennig, Stuart M. Allan, Mark T. Nelson, Adam S. Greenstein, Harry A. T. Pritchard

**Affiliations:** ^a^Division of Cardiovascular Sciences, School of Medical Sciences, Faculty of Biology, Medicine and Health, University of Manchester, Manchester M13 9PL, United Kingdom; ^b^Geoffrey Jefferson Brain Research Centre, The Manchester Academic Health Science Centre, Northern Care Alliance National Health Service Foundation Trust, University of Manchester, Manchester M13 9PL, United Kingdom; ^c^Division of Neuroscience, School of Biological Sciences, Faculty of Biology, Medicine and Health, Manchester Academic Health Science Centre, The University of Manchester, Manchester M13 9PL, United Kingdom; ^d^Department of Pharmacology, Larner College of Medicine, University of Vermont, Burlington, VT 05405; ^e^Manchester University Teaching Hospitals National Health Service Foundation Trust, Manchester M13 9PL, United Kingdom

**Keywords:** ion channels, dementia, hypertension, calcium imaging

## Abstract

The deficit in cerebral blood flow (CBF) seen in patients with hypertension-induced vascular dementia is increasingly viewed as a therapeutic target for disease-modifying therapy. Progress is limited, however, due to uncertainty surrounding the mechanisms through which elevated blood pressure reduces CBF. To investigate this, we used the BPH/2 mouse, a polygenic model of hypertension. At 8 mo of age, hypertensive mice exhibited reduced CBF and cognitive impairment, mimicking the human presentation of vascular dementia. Small cerebral resistance arteries that run across the surface of the brain (pial arteries) showed enhanced pressure-induced constriction due to diminished activity of large-conductance Ca^2+^-activated K^+^ (BK) channels—key vasodilatory ion channels of cerebral vascular smooth muscle cells. Activation of BK channels by transient intracellular Ca^2+^ signals from the sarcoplasmic reticulum (SR), termed Ca^2+^ sparks, leads to hyperpolarization and vasodilation. Combining patch-clamp electrophysiology, high-speed confocal imaging, and proximity ligation assays, we demonstrated that this vasodilatory mechanism is uncoupled in hypertensive mice, an effect attributable to physical separation of the plasma membrane from the SR rather than altered properties of BK channels or Ca^2+^ sparks, which remained intact. This pathogenic mechanism is responsible for the observed increase in constriction and can now be targeted as a possible avenue for restoring healthy CBF in vascular dementia.

## Significance

There are no treatments available for vascular dementia, the second-most common dementia syndrome, and patients decline rapidly after diagnosis. This syndrome is primarily due to hypertension and is associated with reduced cerebral blood flow (CBF). To explore this, we studied mechanisms of vascular dysfunction in pial arteries from hypertensive mice. These mice exhibit reduced CBF, hyperconstricted pial arteries, and behavior approximating human vascular dementia. Using myography, electrophysiology, and Ca^2+^ imaging, we found that the increased constriction was due to separation of the sarcoplasmic reticulum from the plasma membrane in vascular smooth muscle cells, which prevented vasodilatory Ca^2+^ signals from activating large-conductance K^+^ channels. We propose that restoring this coupling could improve CBF and slow disease progression.

Vascular dementia is one of the most devastating sequelae of hypertension. Progressive small vessel disease of the brain results in characteristic radiological changes, including white matter hyperintensities, enlarged perivascular spaces, and cerebral atrophy (1). Clinically, patients with hypertension-induced vascular dementia suffer from depression, gait disturbances, apathy, fatigue, and loss of executive function but often exhibit milder memory loss than that seen in Alzheimer’s disease (2–4). As the illness progresses, however, these latter clinical differences become less delineated, and vascular dementia patients invariably lose all independence and require institutional care. Although the SPRINT (Systolic Blood Pressure Intervention Trial) study showed that intensive blood pressure control reduces incident memory loss (5), parallel studies of vascular dementia indicated that lowering blood pressure alone is not sufficient to prevent or reverse disease progression (6). In this regard, there is a startling void in disease-modifying agents for patients with vascular dementia. Even patients who are experiencing early symptoms but have maintained activities of daily living, a condition termed “mild cognitive impairment”, have no treatment options. Thus, patients with hypertension-induced vascular dementia receive no treatment at all during any stage of their illness, and for most people, the diagnosis heralds an uncertain and justifiably fearful outlook.

The lack of any medical treatment for vascular dementia indisputably reflects our limited understanding of the cellular and molecular mechanisms by which hypertension damages small arteries. It has been established that hypertension triggers remodeling of cerebral small arteries, characterized by a reduction in lumen diameter and an increase in wall thickness (7–10). From a functional perspective, this remodeling likely represents chronic structural changes in the small artery that develop in response to increases in contractility (11–14). At the whole-organ level, however, morphological changes in resistance arteries are reflected in a global reduction in cerebral blood flow (CBF) (15–18) that is associated with an adverse prognosis for patients (19–21).

We recently showed that in the Blood Pressure High (BPH/2) mouse model of polygenic hypertension, function of the endothelial cell inwardly rectifying K^+^ channel, Kir2.1, in brain capillaries is compromised, resulting in neurovascular uncoupling and a reduction in functional hyperemia (22). However, changes in capillary endothelial neurovascular coupling do not fully account for the global reduction in CBF seen in patients with hypertension-induced vascular dementia, pointing to pathology within the pial resistance arteries that traverse the surface of the brain (16).

Pial arteries regulate the flow of blood across the brain surface through myogenic constriction, a mechanism intrinsic to smooth muscle cells that mediates constriction of small resistance arteries in response to increases in intraluminal pressure. The ongoing operation of this constrictive mechanism is fine-tuned by a counterbalancing pathway that promotes vasodilation through activation of large-conductance, Ca^2+^-activated potassium (BK) channels on the plasma membrane (PM) by nearby, localized Ca^2+^-release events (“Ca^2+^ sparks”) from the sarcoplasmic reticulum (SR) of vascular smooth muscle cells (VSMCs) (23). In rat models of hypertension, pial arteries are hyperconstricted due to BK channel dysfunction, providing a plausible explanation for the reduction in CBF (24–26).

Here, we identified the mechanism responsible for increased constriction of pial arteries in the hypertensive BPH/2 mouse model, showing that in hypertensive arterial SMCs, Ca^2+^ sparks occur normally, but the distance between the SR and the BK channels is increased. As a result, Ca^2+^ sparks fail to effectively activate BK channels whose inherent properties remain unchanged. This increased constriction of arteries is associated with a reduction in CBF and behavioral alterations in mice that approximate deficits observed in human vascular dementia patients, including executive dysfunction, apathy, and memory loss.

## Results

### Hypertensive Mice Display Behavioral Alterations Consistent with Vascular Dementia.

**Fig. 1.**
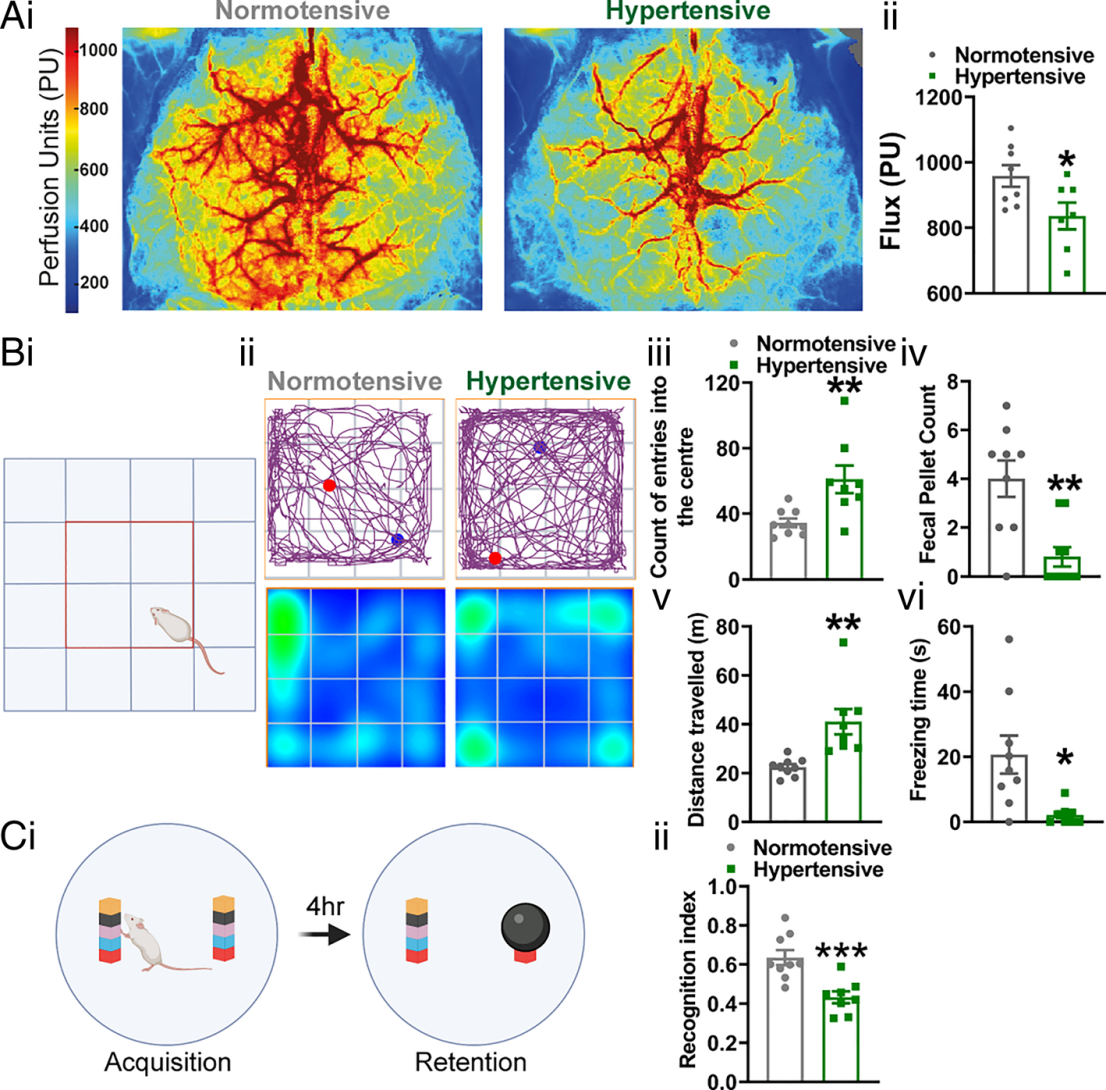
Hypertensive mice display a vascular dementia phenotype. (*A*, *i*) Laser speckle images showing the average perfusion of the dorsal surface of the brain from normotensive (*Left*) and hypertensive (*Right*) mice over a 5-min period. (*A*, *ii*) Average median flux through a surface artery (N = 8 normotensive mice and 7 hypertensive mice; *P < 0.05, unpaired *t* test). (*B, i*) Schematic of the open field maze, with the center shown as a red square. (*B, ii*) *Upper* panels show the pathway traveled by a normotensive (*Left*) and hypertensive (*Right*) mouse, with the blue spot showing the starting position and the red spot showing the final position of the mouse. *Lower* panels show a heat map of the time spent in certain regions of the maze. Summary data showing the number of entries into the center of the maze (*B, iii*), total fecal pellet count (*B, iv*), distance traveled (*B, v*), and time freezing (*B, vi*) in the hypertensive mice compared to the normotensive mice (N = 9 normotensive mice and 8 hypertensive mice; *P = 0.05, **P = 0.01, unpaired *t* test). (*C, i*) Schematic of the novel object recognition test. (*C, ii*) Recognition index for hypertensive mice versus normotensive mice (N = 9 normotensive mice and 8 hypertensive mice; ***P < 0.001, unpaired *t* test).

To determine whether hypertension in the BPH/2 mouse is associated with a reduction in CBF, we performed laser speckle recordings on the exposed, but intact, skull of BPH/2 mice and corresponding Blood Pressure Normal (BPN/3) control mice (hereafter, hypertensive and normotensive mice, respectively) (Fig. 1 *A*, *i*). At 8 mo of age, hypertensive mice displayed approximately a 15% reduction in CBF compared with normotensive mice (Fig. 1 *A*, *ii*).

To determine general well-being, behavioral profiles, and recognition memory, we then conducted cognitive and behavioral tests on 8-month-old hypertensive and normotensive mice. Overall well-being was determined by monitoring and scoring nest building and burrowing behavior, as previously described (27, 28). Our data showed no difference in general well-being between hypertensive and normotensive mice (*SI Appendix*, Fig. S1). In the open-field test, normotensive mice displayed normal thigmotactic behavior, with very few entries into the center of the arena (Fig. 1 *B*, *iii*). In contrast, the number of entries into the center of the arena by hypertensive mice was significantly increased, consistent with a lack of anxiety and diminished awareness of danger (29) (Fig. 1 *B*, *iii*). In support of these behavioral traits, fecal pellet counts were significantly decreased (29) in cages of hypertensive mice compared with those of normotensive mice (Fig. 1 *B*, *iv*). Hypertensive mice also exhibited hyperactivity in the open field arena, as evidenced by increased distance traveled (Fig. 1 *B*, *v*) and decreased freezing time (Fig. 1 *B*, *vi*). The novel object recognition test demonstrated significantly reduced memory in hypertensive mice compared with age-matched normotensive controls (Fig. 1*C*). These data support an association between decreased blood flow to the brain and cognitive dysfunction.

### Impaired VSMC BK Channel Function Augments Arterial Constriction in Hypertension.

**Fig. 2.**
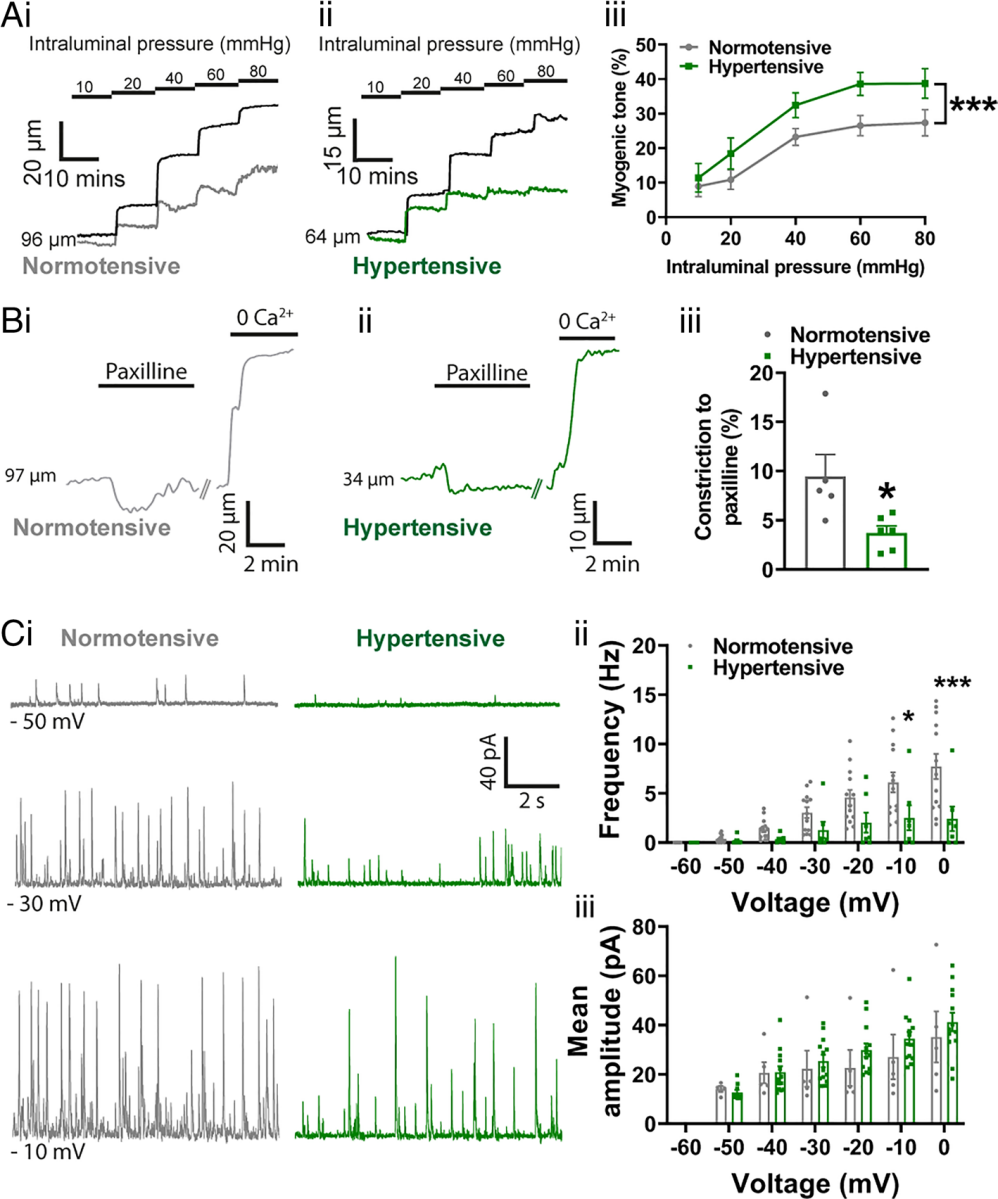
BK channel function is impaired in hypertension. (*A*) Pressure myography traces showing the changes in arterial diameter in response to increasing intraluminal pressure (mmHg) in pial cerebral arteries from a normotensive mouse (*A, i*) and a hypertensive mouse (*A, ii*). Percent myogenic tone was determined by comparing active control of arterial diameter in the presence of Ca^2+^ (normotensive, gray; hypertensive, green) with passive diameter in Ca^2+^-free solutions (black trace). (*A, iii*) Summary data comparing myogenic tone in arteries isolated from hypertensive mice and normotensive mice (n = 14 arteries from 11 normotensive mice and 11 arteries from 9 hypertensive mice; ***P < 0.001, two-way ANOVA). (*B*) Pressure myography experiments showing the change in diameter in response to paxilline (1 μM) in pial arteries isolated from normotensive mice (*B, i*, gray) and hypertensive mice (*B, ii*, green). (*B, iii*) Summary data comparing constriction to paxilline in arteries from hypertensive mice and normotensive mice (n = 5 arteries from 5 normotensive mice and 5 arteries from 5 hypertensive mice; *P < 0.05, unpaired *t* test). (*C, i*) Perforated patch electrophysiological recordings of STOCs in isolated SMCs from pial arteries from normotensive (gray) and hypertensive (green) mice at holding potentials of −50 mV (*Upper*), −30 mV (*Middle*), and −10 mV (*Lower*). Summary data showing STOC frequency (*C, ii*) and amplitude (*C, iii*) in cells isolated from normotensive and hypertensive mouse pial arteries (n = 13 cells from 6 normotensive mice and 7 cells from 4 hypertensive mice; *P < 0.05, ***P < 0.001, two-way ANOVA).

To determine whether the reduction in CBF in hypertensive mice is associated with small artery dysfunction, we measured the diameters of pressurized pial arteries (Fig. 2*A*). Arteries from hypertensive mice showed an increased contractile response to intraluminal pressure, displaying approximately 10% greater constriction across the range of physiological intraluminal pressures (20 to 80 mmHg) (Fig. 2 *A*, *i–iii*). To address a possible role for arterial remodeling, which is considered a key feature of hypertension, we investigated the passive structural properties of cerebral pial arteries using pressure myography but found no differences in wall thickness, cross-sectional area, or wall-to-lumen ratio between hypertensive and normotensive mice (*SI Appendix*, Fig. S2).

To further explore the mechanisms underlying the increase in contractility of resistance arteries, we assessed VSMC BK channel activity. Incubation of pressurized resistance arteries from normotensive mice with the BK channel inhibitor, paxilline, caused approximately a 10% constriction. Constriction to paxilline was reduced by ~60% in arteries from hypertensive mice compared with those from normotensive mice (Fig. 2*B*), indicating a loss of BK channel-mediated control of vascular tone.

BK channels are physiologically activated by Ca^2+^ sparks, and the coupling between these two entities can be measured in isolated VSMCs by recording currents activated by individual Ca^2+^ sparks, referred to as spontaneous transient outward currents (STOCs), using the perforated patch-clamp technique. Consistent with the reduced constriction to paxilline, STOC frequency in SMCs isolated from hypertensive pial arteries was reduced by ~60% across a range of membrane potentials (Fig. 2*C*).

### IK/SK Channel Function and K_IR_ Channel Function Are Preserved in Resistance Arteries from Hypertensive Mice.

To assess whether other principal vasodilatory pathways in pial arteries are altered in hypertensive mice, we examined the function of Kir2.1 inwardly rectifying K^+^ channels, which are expressed in both endothelial cells and SMCs in the cerebral vasculature, as well as endothelial small- and intermediate-conductance Ca^2+^-activated K^+^ channels (SK and IK, respectively). To this end, we exposed pressurized pial arteries to increasing concentrations of external K^+^, which activates Kir2.1 channels, and measured changes in arterial diameter. Low concentrations of K^+^ produced an equivalent vasodilation in both groups, indicating that pial arteries from BPH/2 mice have equivalent Kir2.1 function compared with arteries from BPN/3 mice (*SI Appendix*, Fig. S3). Next, we assessed responses of pressurized pial arteries to the SK/IK channel agonist, NS309. We observed comparable vasodilation between groups, indicating that SK/IK channels retain their function in hypertensive mice (*SI Appendix*, Fig. S3).

### Ca^2+^ Sparks Are Unchanged in Arteries from Hypertensive Mice Compared with Those from Normotensive Mice.

**Fig. 3.**
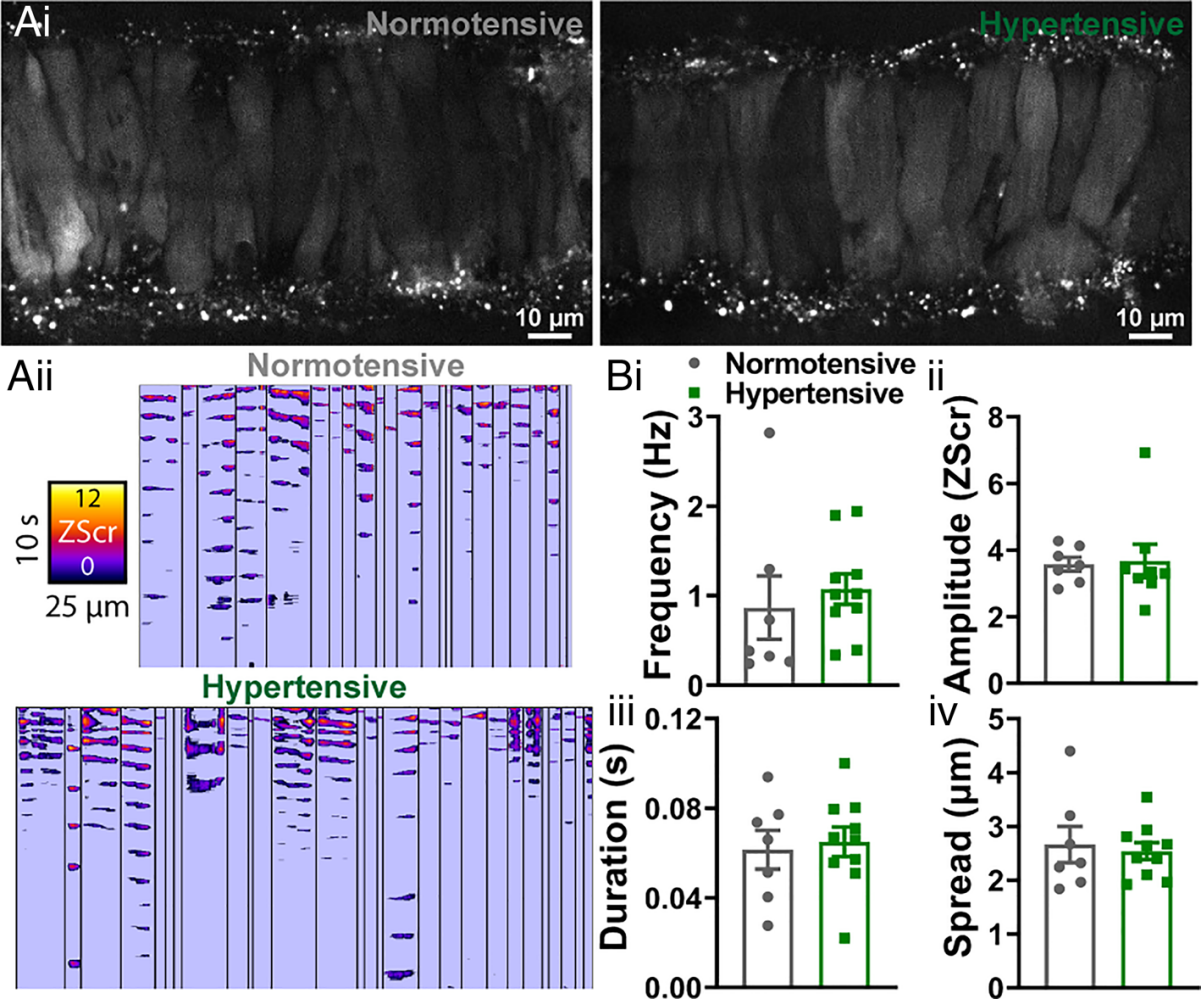
Ca^2+^ spark events are comparable between normotensive and hypertensive vessels. (*A*) Cerebral pial arteries from normotensive and hypertensive mice loaded with Fluo-4-AM, pressurized to 60 mmHg, and imaged using a spinning-disk confocal microscope. (*A*, *ii*) ST maps generated from recordings processed using Volumetry. Events were converted to pixels and given a ZScr to allow categorization into sparks and waves based on duration (length) and spatial spread (width). Summary data showing spark frequency, amplitude, duration, and spread (*B*) in normotensive (gray) and hypertensive (green) mice (n = 7 arteries from 7 normotensive mice and 10 arteries from 10 hypertensive mice; unpaired *t* test).

In the absence of deficits in SK/IK and Kir2.1 vasodilatory pathways in pial arteries from hypertensive mice, we focused on other possible mechanisms that could account for the BK channel dysfunction. A reduced frequency of STOCs could indicate a reduced frequency of Ca^2+^ sparks from the SR, as STOCs are the consequence of Ca^2+^ spark-mediated activation of the BK channel. To test this possibility, we measured Ca^2+^ events in intact, pressurized arteries from normotensive and hypertensive mice. Arteries were loaded with the Ca^2^-indicator dye, Fluo-4-AM (10 μM), and imaged using a high-speed spinning-disc confocal microscope. Videos were processed using an autonomous image analysis approach based on signal-to-noise ratios and statistical Z scores (Zscr), combined with spatiotemporal (ST) mapping, as described previously by our group (30). Briefly, because different areas of a video have a unique background fluorescence, each Ca^2+^ event was converted into pixels and quantified by determining the increase in fluorescence from its own individual baseline. Each pixel was quantified by a Zscr that delineates the average quiescence intensity and SD of the individual event. Once events were identified, they were confirmed as Ca^2+^ sparks based on their functional characteristics, including a duration <0.4 s and a spatial spread of <5 μm within a VSMC. Using this approach, we found no differences in spark frequency or amplitude between the groups (Fig. 3). We also analyzed Ca^2+^ wave events in pressurized cerebral arteries, defined as Ca^2+^-release events that occupy >50% of the cell and have a duration of between 0.5 and 2 s. Like the Ca^2+^ spark analysis, we found no differences in Ca^2+^ wave frequency or amplitude between the groups (*SI Appendix*, Fig. S4).

### BK Channel Properties Are Preserved in Arteries from Hypertensive Mice.

**Fig. 4.**
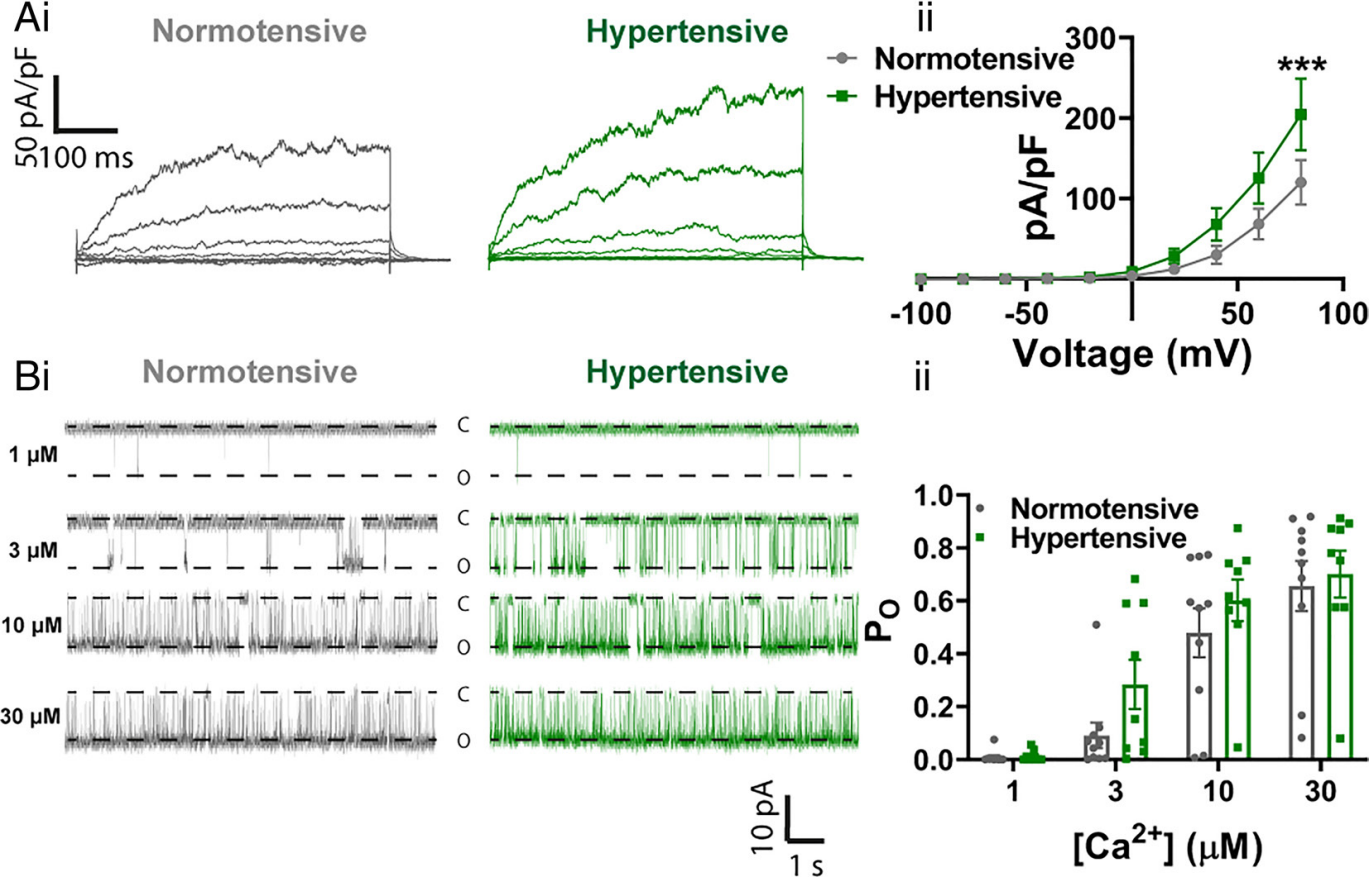
No decrease in functional BK subunit expression in hypertension. (*A*, *i*) Whole-cell ruptured-patch electrophysiological recordings of paxilline-sensitive currents obtained between −100 and 100 mV in SMCs isolated from pial arteries from normotensive (gray) and hypertensive (green) mice. (*A*, *ii*) Summary data comparing BK current density in cells isolated from hypertensive mice and normotensive mice (n = 14 arteries from 5 normotensive mice and 11 arteries from 4 hypertensive mice; *P < 0.05, **P < 0.01, two-way ANOVA). (*B*, *i*) Single-channel inside-out BK channel recordings at −40 mV in exercised patches from SMCs isolated from normotensive (gray) and hypertensive (green) pial arteries. Openings were evoked by exposing patches to 1 μM (*Upper*), 3 μM (*Upper*-*Middle*), 10 μM (*Lower* *Middle*), and 30 μM (*Lower*) free Ca^2+^. (*B*, *ii*) Summary data comparing the open probability (P_O_) between groups at different Ca^2+^ concentrations (n = 10 cells from 4 normotensive mice and 9 cells from 5 hypertensive mice; two-way ANOVA).

To determine whether the lack of BK channel activity in the context of normal Ca^2+^ spark activity was due to altered BK channel properties (e.g., reduced current density or Ca^2+^ sensitivity), we performed additional patch-clamp experiments. For assessment of BK channel current density, BK channels were activated in a whole-cell ruptured-patch configuration by clamping [Ca^2+^]_i_, achieved by including 500 nM Ca^2+^ in the pipette solution. Once this solution had dialyzed into the cell, a step protocol was used to evoke a whole-cell K^+^ current across a large voltage range (−100 to +80 mV, 20 mV increments). The BK channel current density, determined by subtraction of the current in the presence of paxilline (1 µM), was increased at +80 mV but was unchanged at more physiological membrane potentials (−60 to −20 mV) (Fig. 4*A*). To determine Ca^2+^ sensitivity of the BK channel, we performed inside-out single-channel patch-clamp experiments at −40 mV in VSMCs. The apparent Ca^2+^ sensitivity of the BK channel is enhanced by association with an accessory β1 subunit (31). Notably, it has been reported that the β1 subunit is down-regulated in diseases, such as angiotensin 2-induced hypertension (24), which leads to functional uncoupling of Ca^2+^ sparks from BK channels. Our single-channel analysis showed that increasing cytoplasmic [Ca^2+^] (1 to 30 µM) increased channel open probability, as expected, but revealed no differences in Ca^2+^ sensitivity or open probability between cells isolated from pial arteries of normotensive and hypertensive mice (Fig. 4*B*).

### Breakdown in the Integrity of Peripheral Coupling Sites (PCS) Accounts for Reduced BK Channel Function.

**Fig. 5.**
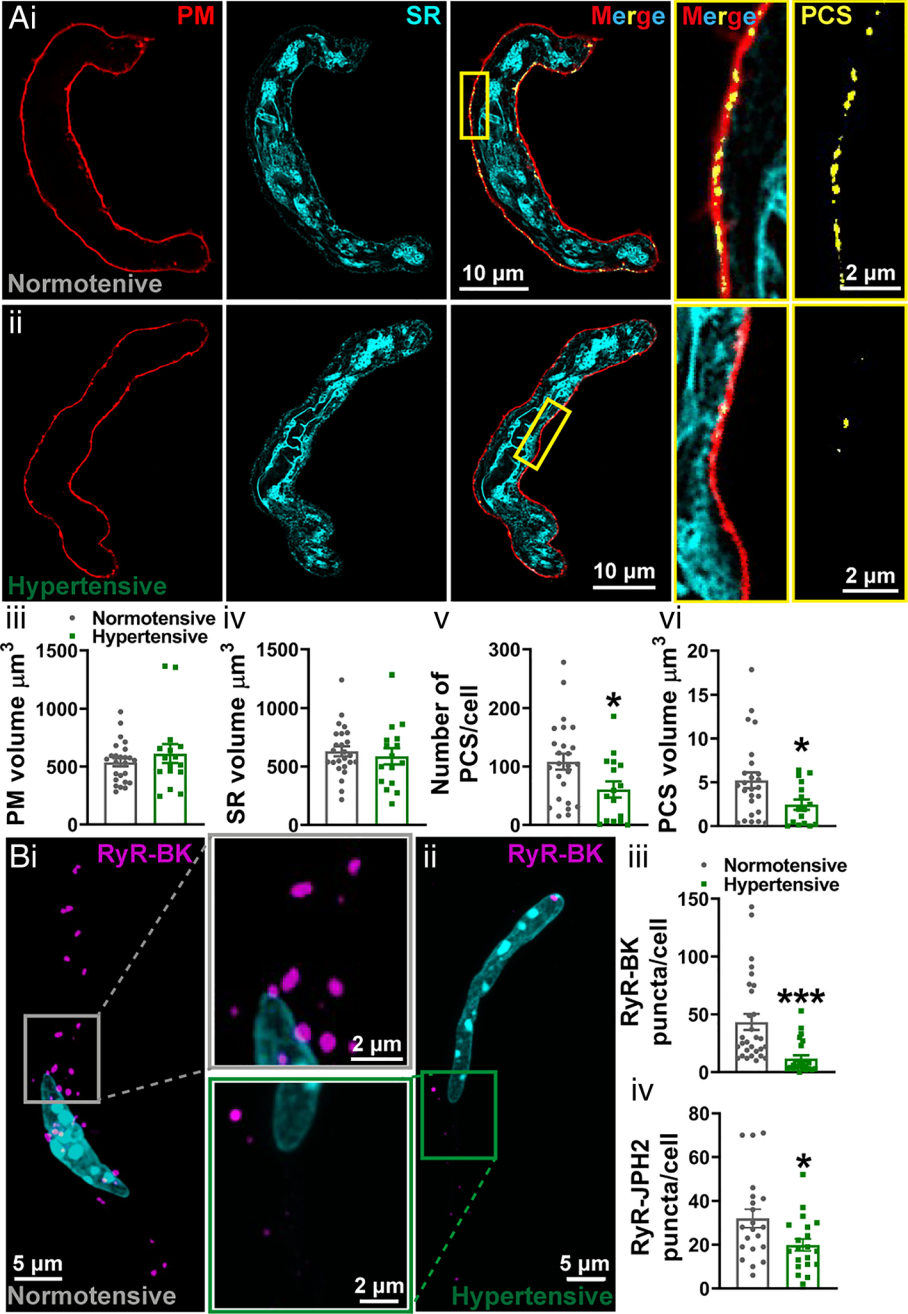
Spatial uncoupling between Ca^2+^ sparks and BK channels in hypertension. (*A*) Live-cell deconvolved confocal imaging of SMCs isolated from normotensive (*A*, *i*) and hypertensive (*A*, *ii*) pial arteries. The PM (red) and SR (cyan) of VSMCs were labeled with specific membrane dyes, with sites of colocalization (yellow) deemed to be PCS. The yellow box is expanded to the right to show the interaction between the two membranes in both merged and PCS only. 3D-reconstructed cells showing the PM volume (*A*, *iii*) and SR volume (*A*, *iv*) and number of PCS (*A*, *v*) and mean PCS volume (*A*, *vi*) per cell (n = 25 cells from 4 normotensive mice and 16 cells from 4 hypertensive mice; *P < 0.05, unpaired *t* test). (*B*) Maximum projection images of proximity ligation assay experiments in isolated SMCs from normotensive (*B*, *i*) and hypertensive (*B*, *ii*) pial arteries, labeled with primary antibodies against RyRs and BK channels. Positive association (<40 nm apart) of the two channels results in fluorescent puncta (magenta). (*B*, *iii*) Summary data showing the number of RyR-BK puncta per cell in SMCs isolated from normotensive and hypertensive mouse pial arteries (n = 29 cells from 6 normotensive mice and 24 cells from 4 hypertensive mice; ***P < 0.001, unpaired *t* test). (*B*, *iv*) Summary data showing RyR–junctophilin (JPH2) puncta per cell in SMCs isolated from hypertensive and normotensive pial arteries (n = 21 cells from 4 normotensive mice and 20 cells from 4 hypertensive mice; *P < 0.05, unpaired *t* test).

The focal increases in Ca^2+^ produced by Ca^2+^ sparks reach micromolar concentrations within the confines of the narrow space separating the ryanodine receptors (RyRs) in the SR membrane that produce them and closely apposed BK channels in the PM. This architectural relationship is thus key to achieving the high local concentrations of Ca^2+^ needed to activate BK channels (32). Accordingly, the absence of changes in Ca^2+^ sparks or BK channel properties points to increased physical separation of RyRs and BK channels as a possible explanation for diminished BK channel activity in our BPH/2 hypertensive mouse model. To visualize and quantify the sites of interaction between the peripheral SR membrane and PM, known as PCS (33), we labeled freshly isolated pial VSMCs with CellMask and ER-Tracker to illuminate the PM and SR, respectively. Fig. 5*A* shows a single-plane, deconvolved confocal microscopy image in which coupling sites are displayed as points of colocalization (yellow) of the PM (red) and SR (cyan). Images were reconstructed to obtain a 3D representation of the cell, from which the number and volume of PCS per cell were calculated (Movies S1 and S2). Although there was no change in the PM or SR volume between the groups (Fig. 5 *A, iii* and *iv*), there was a significant reduction in the number (~45%) and volume (~50%) of PCS within SMCs isolated from pial arteries from hypertensive mice compared with SMCs isolated from pial arteries from normotensive mice (Fig. 5 *A*, *v* and *vi*).

To determine whether there was physical separation between RyRs and BK channels in SMCs from pial arteries of hypertensive mice, we fixed freshly isolated VSMCs and performed proximity ligation assays. Cells were labeled with primary antibodies targeting RyRs and BK channels and with corresponding secondary antibodies containing short complementary oligonucleotide sequences that bind each other if the targets are located within ~40 nm of each other. These oligonucleotide sequences were then amplified and detected as puncta using confocal microscopy (Movies S3 and S4). Maximum intensity projections of VSMCs isolated from normotensive and hypertensive mice (Fig. 5*B*) show that SMCs isolated from cerebral arteries from hypertensive mice display approximately a 70% reduction in the number of puncta compared with SMCs from cerebral arteries from normotensive mice. We also used proximity ligation assays to determine if this uncoupling was due to a loss of peripheral coupling site structural proteins. To this end, we labeled isolated VSMCs with primary antibodies targeting the RyR and junctophilin-2 or the RyR and stromal interaction molecule 1 (STIM1). These experiments revealed a significant decrease in the number of RyR-junctophilin-2 puncta in SMCs isolated from cerebral arteries of hypertensive mice compared with SMCs from cerebral arteries of normotensive mice (Fig. 5*C*). There was no difference between the groups in the number of RyR-STIM1 puncta (*SI Appendix*, Fig. S5).

## Discussion

In this study, we provide a cellular mechanism to account for the increase in contractility of pial arteries in the BPH/2 mouse model of hypertension-related vascular dementia, thus laying the ground for subsequent interventional approaches to rescue arterial homeostasis and restore healthy CBF. We provide important evidence in support of the disease model, confirming that this chronically hypertensive mouse develops a reduction in CBF and vascular dementia-like behavioral alterations. We further show that pial arteries, which traverse the surface of the brain and regulate CBF, are hyperconstricted due to diminished activity of VSMC-hyperpolarizing BK channels, which are activated by brief, RyR-mediated Ca^2+^-release events from the VSMC SR (34). Our data show that in hypertension, the distance between the origin of the Ca^2+^ spark and the BK channel is increased such that the local Ca^2+^ concentration in the vicinity of the BK channel is insufficient to activate the channel.

To put our findings into context, it is important to consider that pial arteries constrict to elevated blood pressure, a mechanism that serves to maintain consistent blood flow to the brain. The diameter of these resistance arteries reflects a balance between this inherent pressure-induced constriction and Ca^2+^ spark-BK channel–mediated dilation (34). Ca^2+^ sparks are generated within VSMCs in response to increases in intraluminal pressure (35), and their close proximity to BK channels results in channel activation, hyperpolarization, and a reduction in the overall level of constriction (35, 36). Thus, at the whole-organ level, CBF is determined by the degree of pial artery constriction.

In this study, the predominant defect in pial arteries from hypertensive mice was increased constriction secondary to spatial uncoupling of Ca^2+^ sparks from the BK channel in VSMCs. In this regard, we have previously shown that VSMC RyRs, which release Ca^2+^ from the SR (optically detected as a Ca^2+^ spark), must be within 20 to 40 nm of plasmalemmal BK channels to generate a detectable BK channel current, measured as a STOC (33, 37). Previous studies have shown that if this distance is exceeded, for example, through damage to the cytoplasmic proteins junctophilin-2 and STIM1 (37, 38) or disruption of the microtubule network (33), Ca^2+^ sparks are present but are unable to activate the BK channel, resulting in a reduction in arterial diameter. Using proximity ligation assays to quantify the engagement of junctophilin-2 and STIM1 with RyRs within VSMCs, we found significantly fewer coupling sites between junctophilin-2 and RyR in hypertensive mice but saw no difference in STIM1-RyR coupling between normotensive and hypertensive mice. Thus, our data suggest that hypertension compromises the capacity of junctophilin-2 to anchor the SR to the plasma membrane. As a consequence, BK channels become separated from RyRs, Ca^2+^ sparks are present but no longer able to elicit a vasodilatory hyperpolarization, and the steady state pressure-induced constriction of arteries is increased. Reinforcing this concept, Ca^2+^ spark frequency, kinetics, and spatial spread were unchanged between normotensive and hypertensive mice. Further exploration of the mechanism by which elevated intraluminal pressure compromises the relationship between junctophilin-2 and RyRs must now be a priority, with the eventual aim of restoring the integrity of their coupling to maintain healthy CBF. There is precedent for this approach. In cardiomyocytes, coupling between the SR and the plasma membrane is a dynamic relationship characterized by formation and dissolution of junctions under the control of microtubule-associated trafficking proteins (39).

Conceptually, however, it is notable that a nanometer-scale shift in the intracellular position of RyR relative to juxtaposed BK channels is associated with such a profound change in CBF and even more remarkably is associated with the development of dementia. BK channels have a low affinity for Ca^2+^ at physiological membrane potentials (−40 mV), requiring 10 to 50 µM Ca^2+^ for significant activation (32). Thus, activation of BK channels requires proximity to Ca^2+^ sparks, making this activation process exceedingly sensitive to uncoupling from the SR through physical separation. A previous study modelling cytoplasmic Ca^2+^ signal dispersal and buffering suggests that following Ca^2+^ entry into the cytoplasm, the signal could drop from a source [Ca^2+^] of 20 µM, to as low as 2 µM at a distance of 40 nm, which is too low for efficient BK channel activation (40). Our proximity ligation assay showed significantly fewer BK–RyR coupling sites with a sub-40-nm spatial separation in VSMCs from hypertensive mice compared with normotensive mice, implying a degree of physical separation in BPH/2 mice sufficient to reduce [Ca^2+^] to less than 10 µM and impair BK channel activation. Our proximity ligation assay data may even be an underestimation of the separation between RyR and BK channels since some proteins that are sufficiently close to each other to produce signals in proximity ligation assays, such as those between 30 and 40 nm apart, would be functionally uncoupled due to the low Ca^2+^ concentration (<10 µM). Future approaches will utilize technologies with greater resolution to quantify this distance more accurately.

In the current study, we found no difference in the Ca^2+^ sensitivity of VSMC BK channels between hypertensive and normotensive mice, obtaining values similar to those found by other groups using healthy C57BL/6J mice (41). With the caveat that a different species was used, our work contrasts with previous studies showing that the Ca^2+^ sensitivity of the BK channel was significantly lower in two rat models with elevated blood pressure (Wistar Kyoto and Spontaneously Hypertensive) than in the normotensive Sprague Dawley rat (42). Ca^2+^ sensitivity of the pial artery SMC BK channel is similarly reduced in an Angiotensin II–induced hypertensive rat model, in this case due to internalization of BK channel subunits (43). Ca^2+^ sparks have been demonstrated in human cerebral arteries (44), so albeit challenging, a priority must be now to examine the Ca^2+^ spark-BK channel vasodilatory pathway in human hypertension.

We have also recently described an increase in pressure-induced constriction in mice with Alzheimer’s disease (APP23 model) secondary to overexpression of amyloid precursor protein (45). Similar to the case for BPH/2 mice, pial arteries from APP23 mice are hyperconstricted due to BK channel dysfunction; however, the pathology upstream from the BK channel diverges from that observed by us in the BPH/2 mouse. Specifically, in the hypertensive BPH/2 mouse, BK channel dysfunction stems from spatial uncoupling of the SR from the PM, whereas the defect in the APP23 mouse reflects a reduction in Ca^2+^ spark frequency.

A further difference in the pattern of vascular pathologies between hypertensive BPH/2 and APP23 mice relates to the function of the endothelial Kir2.1 channel. In all models of Alzheimer’s disease studied to date, there is a reduction in brain endothelial Kir2.1 channel function. This manifests as a failure to vasodilate in response to exogenously applied K^+^ at the pial artery level (45, 46) and neurovascular uncoupling at capillary and arteriole levels (47). However, although we previously demonstrated Kir2.1 dysfunction leading to neurovascular uncoupling in capillary endothelial cells in the BPH/2 model (22), in pial arteries, Kir2.1 function is preserved, as evidenced by the normal dilation to external K^+^ reported here.

The hypertensive mice in this study exhibited a syndrome consistent with the “subcortical” cognitive–affective phenotype of vascular dementia in humans. Most notably, there were significant deficiencies in the novel object-recognition test, consistent with learning deficits. Patients with vascular dementia also exhibit broader symptoms, such as loss of executive function, apathy, and impaired awareness of surroundings (2–4), leading to concerns about their safety in the home and disorientation in new environments. We observed similar symptoms in the BPH/2 mice. Normal responses for mice in the open field test are fear and anxiety, as they are in any unfamiliar environment. Consequently, mice tend to remain close to the walls of the test chamber (thigmotaxis) and generate increased numbers of fecal pellets (29). These behaviors also reflect the conflicting and complex urges of the animal to explore the novel environment and escape from the test chamber to avoid danger. While normotensive mice exhibited appropriate thigmotactic behavior, hypertensive mice spent a much greater proportion of their time in the center of the field test chamber and generated fewer fecal pellets. This could indicate a degree of apathy in the hypertensive mice, but also signifies a broader cognitive failure that represents an inability to detect a threatening environment—an attribute commonly seen in patients with dementia and indeed one that often triggers placement in a safer environment, such as a residential or nursing care home.

Patients with hypertension-induced vascular dementia exhibit deficits in both functional hyperemia and CBF (16, 21). Our findings position us to account for both deficits, with the usual caveats about extrapolating from a mouse model. Thus, according to our data, compromised functional hyperemia is due to defective capillary endothelial Kir2.1 channel function, whereas the reduction in blood flow is attributable to spatial uncoupling of the SR and PM in VSMCs, leading to failure of Ca^2+^ sparks to activate BK channels. Loss of BK channel-mediated membrane potential hyperpolarization in the setting of moment-to-moment pressure-induced constriction is associated with a reduction in CBF. Patients with early vascular dementia or mild cognitive impairment—the forerunner of this condition—will almost certainly have had hypertension-induced small vessel disease of the brain for several years prior to the clinical diagnosis (48). The established damage to small artery function manifests as impaired functional hyperemia and diminished CBF, which may account for the failure of blood-pressure–lowering approaches to restore memory. As such, it is tempting to speculate that a dual approach—lowering of blood pressure in tandem with attempts to reverse small artery pathologies, possibly by protecting the integrity of coupling between RyRs and junctophilin 2—might be a more effective means for slowing disease progression.

## Methods

Procedures used in this study were approved by The University of Manchester Animal Welfare Ethical Review Board and conducted in accordance with UK Home Office Guidance on the Operation of the Animals (Scientific Procedures) Act 1986. Spontaneously hypertensive mice (BPH/2) and their normotensive counterparts (BPN/3) were originally sourced from Jackson Labs, Florida, before breeding was undertaken at the University of Manchester. 8 month-old male BPH/2 and age-matched BPN/3 mice were used throughout this study. Mice had free access to food and water and were housed in pathogen-free conditions under a 12-h day/night cycle, with behavioral assessments undertaken in the day cycle. Laser-doppler blood flow measurements were carried out in anesthetized mice with isoflurane. For pressure myography, imaging, and electrophysiology experiments, mice were killed by overdose of CO_2_ followed by exsanguination. Cerebral pial resistance arteries were dissected from the brain and studied using pressure (isobaric) myography and high-speed spinning-disk confocal microscopy. Arteries were enzymatically digested, and SMCs were studied using patch-clamp electrophysiology and confocal imaging. Full details of all materials and methods can be found in *SI Appendix*.

## Supplementary Material

Appendix 01 (PDF)

Movie S1. Animated representation of a cerebral artery SMCs isolated from a normotensive mouse, labeled with live-cell membrane dyes and imaged using deconvolved confocal microscopy. Images were reconstructed and rendered to show the PM (red), SR (cyan), and peripheral coupling sites (PCS; yellow).

Movie S2. Animated representation of a cerebral artery SMCs isolated from a hypertensive mouse, labeled with live-cell membrane dyes and imaged using deconvolved confocal microscopy. Images were reconstructed and rendered to show the PM (red), SR (cyan), and peripheral coupling sites (PCS; yellow).

Movie S3. Animated representation of a cerebral artery SMCs isolated from a normotensive mouse, showing positive proximity ligation assay puncta (magenta).

Movie S4. Animated representation of a cerebral artery SMCs isolated from a hypertensive mouse, showing positive proximity ligation assay puncta (magenta).

## Data Availability

All study data are included in the article and/or supporting information.
