# Supplementary material for: Uncoupling of Ca2+ sparks from BK channels in cerebral arteries underlies hypoperfusion in hypertension-induced vascular dementia
Source: Proc Natl Acad Sci U S A. 2023 Aug 7;120(33):e2307513120. doi: 10.1073/pnas.2307513120 (PMC10433456; doi:10.1073/pnas.2307513120)
Supplement: Supplementary file 1 — Appendix 01 (PDF) [file pnas.2307513120.sapp.pdf]

PNAS

Supporting Information for

Uncoupling of Ca^{2+} sparks from BK channels in cerebral arteries underlies hypoperfusion in hypertension-induced vascular dementia.

Jade L. Taylor[1,2], Katy R. Walsh[1,2,3], Ioana-Emilia Mosneag[2,3], Thea G.E Danby[1,2], Nadim Luka[2,3], Bishal Chanda[2,3], Ingo Schiessl[2,3], Ross A. Dunne[2], David Hill-Eubanks[4], Grant W. Hennig[4], Stuart M. Allan[2,3], Mark T. Nelson[1,2,4], Adam S. Greenstein[1,2] & Harry A.T. Pritchard[1,2]

[1]Division of Cardiovascular Sciences, Faculty of Biology, Medicine and Health, University of Manchester & Manchester University Teaching Hospitals NHS Foundation Trust, Manchester, United Kingdom

[2]Geoffrey Jefferson Brain Research Centre, The Manchester Academic Health Science Centre, Northern Care Alliance NHS Foundation Trust, University of Manchester, UK

[3]Division of Neuroscience, School of Biological Sciences, Faculty of Biology, Medicine and Health, Manchester Academic Health Science Centre, The University of Manchester, Oxford Road, Manchester, UK

[4]Department of Pharmacology, Larner College of Medicine, University of Vermont, Burlington, VT, USA

Mark Nelson, Department of Pharmacology, Larner College of Medicine, University of Vermont, Burlington, VT, USA. Mark.nelson@uvm.edu

This PDF file includes:

Supporting text
Figures S1 to S5
Legends for Movies S1 to S4
SI References

Other supporting materials for this manuscript include the following:

Movies S1 to S4

Supporting Information Text

Supplementary Methods

Animal studies

All procedures used in this study were approved by The University of Manchester Animal Welfare Ethical Review Board and were conducted in accordance with UK Home Office Guidance on the Operation of the Animals (Scientific Procedures) Act 1986. Spontaneously hypertensive mice (BPH/2) and their normotensive counterparts (BPN/3) were originally sourced from Jackson Labs, Florida, before breeding was undertaken at the University of Manchester. Eight-month-old male BPH/2 and age-matched BPN/3 mice were used throughout this study. Mice had free access to food and water and were housed in pathogen-free conditions under a 12 h day/night cycle.

Laser-Doppler blood flow measurements

Mice were anesthetized with 4% isoflurane (Abbott, Berkshire, UK) and secured in a stereotaxic frame (World Precision Instruments, USA) positioned under a Moor FLPI2 Laser Speckle Imager (Moor Instruments, UK). Isoflurane anesthesia was maintained at 1.5% throughout imaging, and body temperature was maintained at 37°C using an electric heating pad and monitored using an anal thermometer. The skull was exposed by dissecting the scalp along the midline, and the region was cleared for imaging by securing the skin on top of the skull using surgical clips. An ultrasound gel was applied to the mouse skull and a glass coverslip was mounted on top to improve imaging quality. Laser speckle cranial imaging was conducted for 10 min with a frame rate of 1500/min. Following imaging, mice were euthanized by cervical dislocation. A region of interest was drawn around the same middle cerebral artery branch in each image using Moor FLPI2 software (Axminster, UK), and the median flux was averaged over the last 5 min of the recording. (Readings fluctuated during the first 5 min before animals reached a steady state of anesthesia.)

Behavioral assessments

Behavior was assessed during the light phase (7 am to 7 pm) unless otherwise stated. After assigning to cages according to group, mice were individually handled on three consecutive days before testing. All tests were performed after a minimum of 1 h acclimatization to the experimental room. Each mouse received an individual code, and experimenters were blinded for analysis of behavioral tests, except for manual video scoring for the novel object-recognition test. In the latter, the genotype of the mice could be distinguished due to their different fur coloring—white for BPH/2 and brown for BPN/3 animals.

Nesting: Spontaneous, innate nesting behavior was assessed by first placing mice in individual cages containing 20 g of Sizzle-Nest material (Datesand LTD, Manchester, UK) 1 h before the dark phase and then leaving them overnight with free access to food and water. Nests were scored the next day during the light cycle by two independent assessors. Scoring was performed as previously

described (1). Briefly, nests were scored as follows: 0, material not manipulated; 1, no clear nest site (majority of nesting material not manipulated to one cage quadrant); 2, nest present but flat; 3, nest present with raised walls <30 mm in height; 4, nest present with walls 31-49 mm in height; and 5, nest present with walls > 50 mm in height.

Burrowing: As an additional assessment of well-being, two burrowing tests were conducted at least 48 h apart. In these tests, mice were singly housed for 2 h, and 150 g of standard diet food pellets (Envigo, UK) were added inside custom-made burrowing tubes (200-mm lengths of 68 -mm diameter PVC downpipe) as previously described (2, 3). Thereafter, the quantity of food pellets burrowed was recorded and mice were returned to their home cages.

Open field: For open-field tests, mice were individually placed in a square Perspex arena (450 × 450 × 200 mm) and allowed to explore freely for 5 min. Video recordings were obtained from a camera suspended above the arena and automatically analyzed using ANY-Maze v7.10 software (Stoelting, USA). The following parameters were automatically calculated: distance travelled, number of entries and time spent in the center zone, and freezing time (1-s threshold). Fecal pellet droppings were also counted at the end of the test.

Novel object recognition: The novel object recognition (NOR) test was used to assess short-term memory. A round arena, 30 cm in diameter, was fitted with two identical objects functioning as familiar objects for habituation and acquisition phases. A novel object was randomly assigned for the retention phase. Objects were created with Lego pieces and cleaned between each use. After habituating to the arena as a cage-based group, mice were subject to a 5-min acquisition period, followed by a 5-min retention test 4 h later. Active exploration was defined as touching, sniffing, or exploring the objects. Each phase was video recorded from above, and object-interaction time was manually timed. Recognition index was calculated using the formula, TN/(TN+TF), where TN is the time spent exploring the novel object and TF is the time spent exploring the familiar object, as previously described (4).

Pressurized arteries

For pressure myography, imaging and electrophysiology experiments, mice were euthanized by overdose of CO_2 followed by exsanguination. The brain was removed and kept in ice-cold physiological saline solution (Mg-PSS; 140 mM NaCl, 5 mM KCl, 2 mM $MgCl_2$, 10 mM HEPES and 10 mM glucose; pH adjusted to 7.4 with 1 M NaOH) before conducting experiments. Cerebral pial resistance arteries were dissected from the brain and stored in Mg-PSS on ice.

Cerebral pial (posterior cerebral or superior cerebellar) arteries were dissected from the brain and stored in chilled Mg-PSS. Arterial segments were mounted on glass pipettes of a similar size in an

arteriograph chamber (Living Systems Instrumentation, VT). Arteries were allowed to equilibrate for 15 min in pre-warmed (37°C) physiological saline solution (PSS; 125 mM NaCl, 3 mM KCl, 26 mM $NaHCO_3$, 1.25 mM $NaH_2PO_4 \cdot H_2O$, 1 mM $MgCl_2$, 4 mM glucose and 2 mM $CaCl_2$, bubbled with 5% CO_2 in biological air) before being pressurized to 60 mmHg. Vessels were allowed to develop myogenic tone before using in either pressure-response or paxilline experiments. Luminal diameter was continuously measured throughout the experiment using a camera and edge-detection software (IonOptix). Percentage constriction was calculated as the degree of change relative to passive diameter using equation: (baseline diameter – constricted diameter)/passive diameter x 100. Percentage dilation was calculated as the full dilation for each vessel: (active diameter – baseline)/(passive diameter – baseline) x 100. Myogenic tone was calculated as (passive lumen diameter – active lumen diameter/passive lumen diameter) x 100. Passive diameter was determined by exposing arteries to a Ca^{2+}-free PSS solution with the composition, 119 mM NaCl, 4.7 mM KCl, 21 mM NaHCO3, 1.18 mM KH2PO4, 1.17 mM MgSO4, 3 mM EGTA, 4 mM glucose and 0.01 mM diltiazem.

Ca^{2+} imaging in pressurized arteries: Cerebral pial arteries from BPH/2 or BPN/3 animals were dissected out and placed in Mg-PSS solution containing the Ca^{2+} indicator dye, Fluo-4AM (10 µM), and pluronic acid (0.05%), and allowed to stand at room temperature in the dark for 30 min. After incubation with Fluo-4AM, arteries were mounted onto glass micropipettes in an arteriography chamber and superfused with a Ca^{2+}-imaging physiological saline solution (CaIPSS; 125 mM NaCl, 3 mM KCl, 26 mM $NaHCO_3$, 1.25 mM $NaH_2PO_4 \cdot H_2O$, 1 mM $MgCl_2$, 4 mM glucose and 2 mM $CaCl_2$), aerated with 5% CO_2/21% O_2 (balance N_2) and warmed to 37°C. After a 20-min equilibration period at 5 mmHg, arteries were pressurized to 60 mmHg. Thereafter, Ca^{2+} events were imaged (excitation wavelength, 488 nm; fluorescence emission collected above 510 nm) using a high-speed spinning-disc confocal microscope (Nikon Eclipse TE-2000U). Images (512 × 512 pixels; field of view, 131 × 131 µm) were recorded every 18.9 ms (53 fps) using a 60× water-immersion objective (final magnification, 600×; NA1.2).

Ca^{2+} events were analyzed as previously described (5). In brief, videos of Ca^{2+} events were imported into custom-written software (Volumetry G9e; G.W.H) before being motion-stabilized and normalized with respect to background intensity. Because basal fluorescence intensity varies across VSMCs, this analysis records basal fluorescence at quiescence (SDq) and gives a Z-score (Zscr) to increases from this value. A threshold is applied to the image such that only sufficiently large increases in Zscr are defined as Ca^{2+} events. A range of quantitative metrics were extracted from the videos, including duration, size and direction of spread, and maximum intensity (Zscr). Using these data, we defined Ca^{2+} sparks as signals with a duration of less than 0.4 seconds and

a spatial spread less than 5 µM, and Ca^{2+} waves as events with a duration of 0.5-2 s and a spatial spread covering more than 50% of the cell.

VSMC isolation

VSMCs were isolated by digesting cerebral pial arteries in Mg-PSS supplemented with papain (1.0 mg/ml; Worthington Biochemical, NJ, USA), dithioerythritol (1 mg/ml) and BSA (10 mg/ml) at 37°C for 12 min, washed three times with Mg-PSS, and then incubated a second time for 14 min at 37°C in type II collagenase (1.0 mg/ml; Worthington). VSMCs were then liberated by triturating digested arteries, stored in ice-cold Mg-PSS, and studied within 6 h.

Electrophysiology

Currents were recorded using an AxoPatch 200B amplifier equipped with an Axon CV 203BU headstage (Molecular Devices), filtered at 1 kHz, digitized at 40 kHz, and stored for subsequent analysis. Clampex and Clampfit (version 10.2; Molecular Devices) were used for data acquisition and analysis, respectively. All recordings were performed at room temperature (~22°C). VSMCs were transferred to a recording chamber and allowed to adhere to glass coverslips for 10 min at room temperature. Recording electrodes (5–7 MΩ) were pulled and polished.

STOCs were recorded using the perforated-patch whole-cell technique, in which amphotericin B (40 µM) is included in the pipette solution to allow electrical access. Perforation was deemed acceptable if series resistance was less than 40 MΩ. Spontaneous transient outward currents (STOCs) were recorded in a bathing solution containing 134 mM NaCl, 6 mM KCl, 1 mM $MgCl_2$, 2 mM $CaCl_2$, 10 mM Hepes, and 10 mM glucose at pH 7.4 (NaOH). The pipette solution contained 110 mM K-aspartate, 1 mM $MgCl_2$, 30 mM KCl, 10 mM NaCl, 10 mM Hepes, and 5 µM EGTA at pH 7.2 (NaOH). For STOC recordings, VSMCs were voltage-clamped (for 30 s) at a range of membrane potentials (-60 to 0 mV). STOCs were analyzed using the threshold method, and STOC frequency was determined at each membrane potential.

Whole-cell K^+ currents were recorded in the ruptured whole-cell configuration using a step protocol (−100 to +80 mV in 20 mV steps for 500 ms) from a holding potential of −30 mV. Whole-cell BK currents were isolated by current subtraction following administration of the selective BK channel blocker, paxilline (1 µM). Current–voltage (I–V) plots were generated using currents averaged over the last 50 ms of each voltage step. The bathing solution consisted of 134 mM NaCl, 6 mM KCl, 10 mM Hepes, 10 mM glucose, 2 mM $CaCl_2$, and 1 mM $MgCl_2$ at pH 7.4 (NaOH). The pipette solution consisted of 140 mM KCl, 1.9 mM $MgCl_2$, 75 µM Ca^{2+} (500 nM free Ca^{2+}), 10 mM Hepes, 0.1 mM EGTA, and 2 mM Na_2ATP at pH 7.2 (KOH). Free Ca^{2+} was determined using WEBMAXC software

(C. Patton, Stanford University Pacific Grove, CA, USA; https://somapp.ucdmc.ucdavis.edu/pharmacology/bers/maxchelator/maxc.html).

Single-channel BK currents were recorded from inside-out membrane patches of isolated pial artery VSMCs at -40 mV. The bathing solution consisted of 140 mM KCl, 10 mM HEPES (pH 7.2 with Tris), 1 mM N-(2-hydroxyethyl)ethylenediamine-N,N′,N′-triacetic acid (HEDTA) containing different free Ca^{2+} concentrations (1–30 µM; calculated in WEBMAXC software). Pipettes were filled with bathing solution supplemented with 10 µM free Ca^{2+}.

Live-cell imaging of the SR and PM

SMCs isolated from BPN/3 and BPH/2 pial arteries were allowed to adhere to glass-bottom 35-mm dishes (Corning; Fisher Scientific) for 1 h on ice. The SR and PM were labeled using a protocol that we previously described(6, 7). VSMCs were treated with ER-Tracker Green (5 µg/mL; Invitrogen) in Mg-PSS for 30 min at 37°C, washed three times with Mg-PSS, and then treated with CellMask Deep Red (5 µg/mL; Invitrogen) for 5 min at 37°C. Fluorescence images were obtained with an Andor Dragonfly 200 spinning-disk upright confocal microscope (Andor Technologies) using a 60× water-immersion objective (N.A. 0.9). Images were collected using a Zyla 4.2 Plus sCMOS camera. Z-stacks were acquired at 0.25-µm steps. Texas Red and fluorescein isothiocyanate were excited by illumination with 647- and 488-nm laser lines, respectively. All images were acquired at 2,048 pixels × 2,048 pixels and deconvolved using FUSION software (version 2.0; Oxford Instruments). Images were further processed and analyzed using Imaris (v.9.2) software (Bitplane). Lateral chromatic aberrations and astigmatisms were determined using fluorescent microbeads (FocalCheck-TetraSpec; Fisher Scientific) and corrected during postprocessing. Deconvolved Z-stack images were imported and reconstructed, and each channel was thresholded. A third (colocalization) channel in which voxels were positive for both the red (PM) and cyan (SR) channel was created, and 3D surface plots were created for the PM, SR, and colocalization channels. Voxel size = 0.048 x 0.048 x 0.250 µm (xyz).

Proximity ligation assay

SMCs isolated from BPN/3 and BPH/2 pial arteries were allowed to adhere to 22 x 22 mm coverslips for 1 h on ice, then fixed with 2% paraformaldehyde for 15 min at room temperature. Thereafter, cells were washed with 1x phosphate-buffered saline (PBS; Sigma-Aldrich), permeabilized with 0.1% Triton X-100 (Sigma Aldrich) for 10 min, and blocked with 50% SEA Block (Abcam) solution in PBS. Cells were incubated overnight at 4°C with primary antibodies against RyR (Thermo Fisher Scientific MA3-916, 1:100), BKα (Alomone Labs APC-021, 1:200), junctophilin-2 (Thermo Fisher Scientific 40-5300, 1:400) or STIM-1 (BD Bioscience 610954, 1:200) diluted in PBS containing 20% SEA Block. After removing excess primary antibody with a series of

washes with 20% SEA Block, the proximity ligation assay (PLA) was carried out using a Duolink in Situ Kit (Sigma-Aldrich) according to the manufacturer's instructions. Briefly, cells were exposed to anti-mouse (negative probe) and anti-rabbit (positive probe) oligonucleotide-tagged secondary antibodies for 1 h at 37°C and washed, followed by a ligation step (30 min at 37°C) that crosslinks antibodies that are within ~40 nm of each other. A specific fluorescent signal was generated by applying an amplification step (100 min at 37°C) to oligonucleotide tags of crosslinked antibodies, after which cells were washed and mounted on coverslips using Duolink In Situ Mounting Medium containing DAPI (4',6-diamidino-2-phenylindol). Cells were imaged using a protocol similar to that for live-cell imaging, employing 647- and 405-nm laser lines to excite Texas Red-PLA probes and DAPI, respectively. Analyses were carried out with Imaris software using the 'spot analysis' function.

Statistical analysis

Data are expressed as means ± standard error of the mean (S.E.M.), with 'n' referring to the number of cells or vessels, and 'N' corresponding to the number of animals in each group. Data were deemed normally distributed and compared as indicated in figure captions using paired or unpaired t-tests or two-way analysis of variance (ANOVA) with a Sidak post hoc test. Statistical testing was performed using GraphPad Prism software (Version 8.4.3; GraphPad software, San Diego, CA, USA). A P-value ≤ 0.05 was considered significant; individual p-value ranges are indicated by stars in figure legends and figure panels.

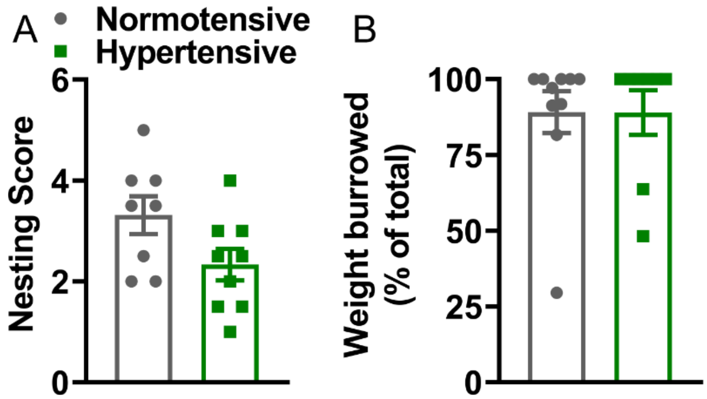

Fig. S1. **Normal general well-being in both normotensive and hypertensive mice**. A) Comparison of overnight nest-building in a clean cage by normotensive and hypertensive mice. B) Comparison of the ability of normotensive and hypertensive mice to burrow through a tube to collect food (N = 9 normotensive mice and 8 hypertensive mice; unpaired t-test).

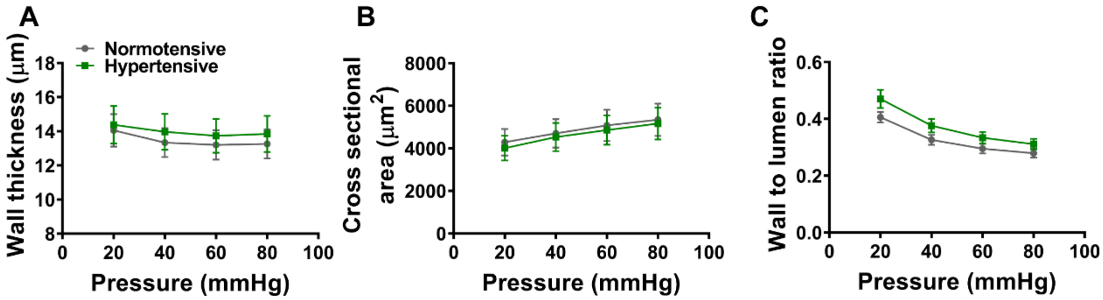

Fig. S2. **Supplemental figure 2: Pial artery wall structure**. Passive diameter recordings in pressurized cerebral arteries were used to determine the structural of the pial vascular wall. Wall thickness (A), cross-sectional area (B) and wall-to-lumen ratio (C) in pial arteries from normotensive and hypertensive mice (N = 14 vessels from 11 normotensive mice and 11 vessels from 9 hypertensive mice; two-way ANOVA).

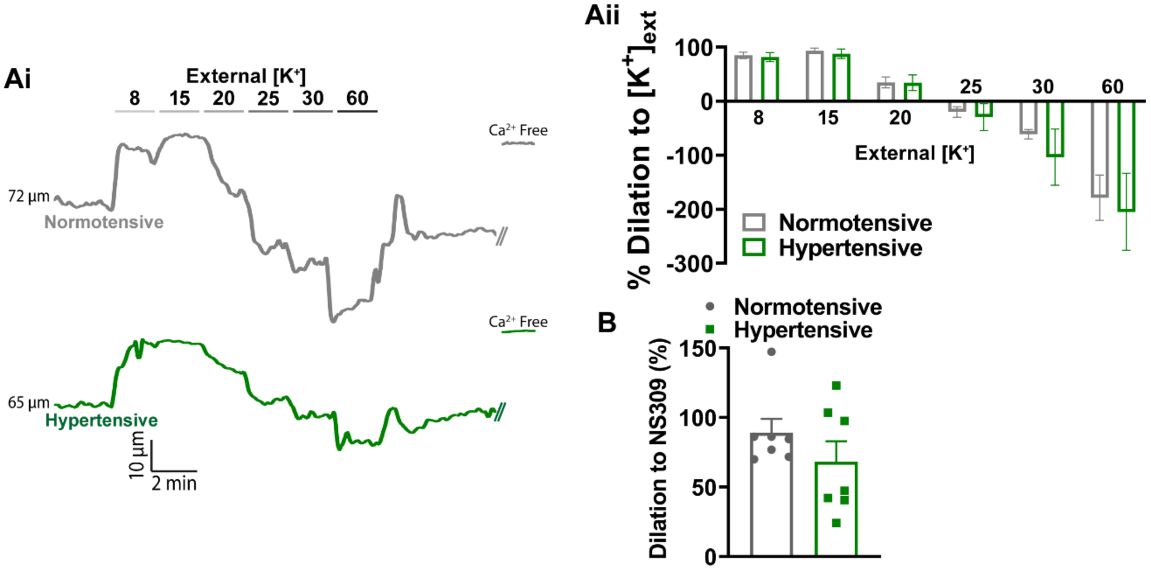

Fig. S3. K_{IR} and IK/SK channels are functionally preserved in hypertensive pial arteries. Ai) Diameter traces of pressurized normotensive (upper, gray) and hypertensive (lower, green) pial arteries exposed to increasing concentrations of extracellular K^+. Vessels developed tone in PSS containing normal K^+ (3 mM) before exposure to increasing concentrations of K^+ (8–60 mM). Aii) Summary data showing the percent vasodilation to increasing external K^+ concentrations ($[K^+]_{ext}$) (n = 7 vessels from 7 normotensive mice and 12 vessels from 8 hypertensive mice; two-way ANOVA). B) Summary data showing the percentage vasodilation to the IK/SK channel opener, NS309, in pial arteries from normotensive and hypertensive mice (n = 7 vessels from 7 normotensive mice and 7 vessels from 6 hypertensive mice; unpaired t-test).

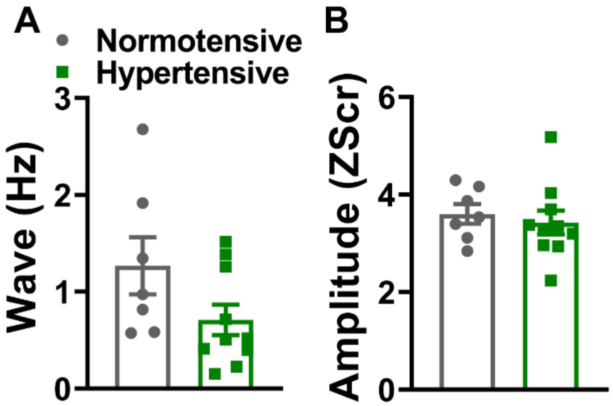

Fig. S4. **Supplemental figure 4: No change in Ca^{2+} wave frequency in hypertension**. The recordings shown in Figure 4 were also analyzed for frequency (A) and amplitude (B) of Ca^{2+} waves, defined as events that occupied more than 50% of the cell and lasted between 0.5 and 2 seconds (N = 7 vessels from 7 normotensive mice and 10 vessels from 10 hypertensive mice; unpaired t-test).

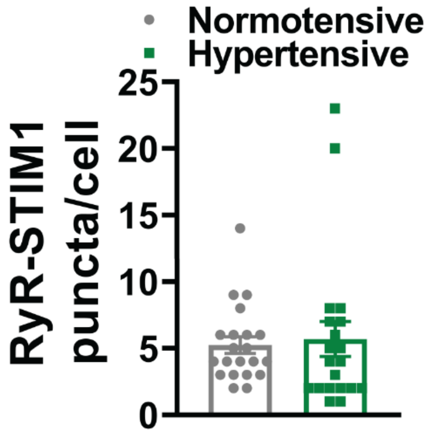

Fig. S5. **Supplemental figure 5: No change in RyR-STIM1 interactions in hypertension.** Summary data showing RyR-STIM1 proximity ligation assay puncta per cell in SMCs isolated from pial arteries of normotensive and hypertensive mice (n = 21 cells from 4 normotensive mice and 20 cells from 4 hypertensive mice; unpaired t-test).

Legends for Movies

Movie S1

Animated representation of a cerebral artery SMCs isolated from a normotensive mouse, labeled with live-cell membrane dyes and imaged using deconvolved confocal microscopy. Images were reconstructed and rendered to show the PM (red), SR (cyan), and peripheral coupling sites (PCS; yellow).

Movie S2

Animated representation of a cerebral artery SMCs isolated from a hypertensive mouse, labeled with live-cell membrane dyes and imaged using deconvolved confocal microscopy. Images were reconstructed and rendered to show the PM (red), SR (cyan), and peripheral coupling sites (PCS; yellow).

Movie S3

Animated representation of a cerebral artery SMCs isolated from a normotensive mouse, showing positive proximity ligation assay puncta (magenta).

Movie S4 Animated representation of a cerebral artery SMCs isolated from a hypertensive mouse, showing positive proximity ligation assay puncta (magenta).

SI References

1. B. N. Gaskill, A. Z. Karas, J. P. Garner, K. R. Pritchett-Corning, Nest building as an indicator of health and welfare in laboratory mice. *J Vis Exp* 10.3791/51012, 51012 (2013).
2. R. Deacon, Assessing burrowing, nest construction, and hoarding in mice. *Journal of visualized experiments : JoVE* 10.3791/2607, e2607-e2607 (2012).
3. C. J. Cunningham *et al.*, Systemic conditioned medium treatment from interleukin-1 primed mesenchymal stem cells promotes recovery after stroke. *Stem Cell Research & Therapy* **11**, 32 (2020).
4. M. Antunes, G. Biala, The novel object recognition memory: neurobiology, test procedure, and its modifications. *Cogn Process* **13**, 93-110 (2012).
5. J. L. Taylor *et al.*, Functionally linked potassium channel activity in cerebral endothelial and smooth muscle cells is compromised in Alzheimer's disease. *Proc Natl Acad Sci U S A* **119**, e2204581119 (2022).
6. H. A. T. Pritchard *et al.*, Microtubule structures underlying the sarcoplasmic reticulum support peripheral coupling sites to regulate smooth muscle contractility. *Sci Signal* **10** (2017).
7. H. A. T. Pritchard *et al.*, Nanoscale coupling of junctophilin-2 and ryanodine receptors regulates vascular smooth muscle cell contractility. *Proc Natl Acad Sci U S A* **116**, 21874-21881 (2019).
